# ^18^F-FDG PET/CT in Infective Endocarditis: Indications and Approaches for Standardization

**DOI:** 10.1007/s11886-021-01542-y

**Published:** 2021-08-07

**Authors:** D. ten Hove, R.H.J.A. Slart, B. Sinha, A.W.J.M. Glaudemans, R.P.J. Budde

**Affiliations:** 1 Department of Nuclear Medicine and Molecular Imaging, University of Groningen, University Medical Center Groningen, Hanzeplein 1, 9713GZ, Groningen, The Netherlands; 2 Department of Microbiology and Infection Prevention, University of Groningen, University Medical Center Groningen, Groningen, The Netherlands; 3 Department of Biomedical Photonic Imaging, Faculty of Science and Technology, University of Twente, Enschede, the Netherlands; 4 Department of Radiology and Nuclear Medicine, Erasmus University Medical Center, Rotterdam, the Netherlands

**Keywords:** Endocarditis, FDG, PET/CT, Indications, Standardization

## Abstract

**Purpose of Review:**

Additional imaging modalities, such as FDG-PET/CT, have been included into the workup for patients with suspected infective endocarditis, according to major international guidelines published in 2015. The purpose of this review is to give an overview of FDG-PET/CT indications and standardized approaches in the setting of suspected infective endocarditis.

**Recent Findings:**

There are two main indications for performing FDG-PET/CT in patients with suspected infective endocarditis: (i) detecting intracardiac infections and (ii) detection of (clinically silent) disseminated infectious disease. The diagnostic performance of FDG-PET/CT for intracardiac lesions depends on the presence of native valves, prosthetic valves, or implanted cardiac devices, with a sensitivity that is poor for native valve endocarditis and cardiac device-related lead infections, but much better for prosthetic valve endocarditis and cardiac device-related pocket infections. Specificity is high for all these indications. The detection of disseminated disease may also help establish the diagnosis and/or impact patient management.

**Summary:**

Based on current evidence, FDG-PET/CT should be considered for detection of disseminated disease in suspected endocarditis. Absence of intracardiac lesions on FDG-PET/CT cannot rule out native valve endocarditis, but positive findings strongly support the diagnosis. For prosthetic valve endocarditis, standard use of FDG-PET/CT is recommended because of its high sensitivity and specificity. For implanted cardiac devices, FDG-PET/CT is also recommended, but should be evaluated with careful attention to clinical context, because its sensitivity is high for pocket infections, but low for lead infections. In patients with prosthetic valves with or without additional aortic prosthesis, combination with CTA should be considered. Optimal timing of FDG-PET/CT is important, both during clinical workup and technically (i.e., post tracer injection). In addition, procedural standardization is key and encompasses patient preparation, scan acquisition, reconstruction, subsequent analysis, and clinical interpretation. The recommendations discussed here will hopefully contribute to improved standardization and enhanced performance of FDG-PET/CT in the clinical management of patients with suspected infective endocarditis.

## Introduction

Infective endocarditis (IE) is a serious condition with substantial morbidity and mortality. While it is relatively rare with an incidence of 3–10 per 100,000 per year [1], there is evidence that this incidence is increasing. This is in part due to an increasing life expectancy, expanding options for cardiac valve repair and/or replacement, and increasing use of cardiac-implanted electronic devices [2–5]. IE is a diagnostic challenge because of its highly variable clinical presentation. The mainstay of diagnosis is based on microbiological evidence (mainly blood cultures) and imaging findings that need to be interpreted in combination with clinical signs. These are scored as either minor or major criteria, and integrated into the modified Duke criteria, resulting in a rejected, possible, or definite diagnosis of IE [6]. It is important to note that the modified Duke criteria have variable sensitivity and specificity, especially in the setting of prosthetic valve endocarditis (PVE) and cardiac device-related endocarditis (CDRIE), and they should support rather than replace clinical judgement [7, 8]. Traditionally, echocardiography has had a central role in establishing the diagnosis. In 2015, both American and European guidelines have included additional imaging modalities [8, 9], with the latter formally including these findings as formal criteria—leading to an amended scoring system (ESC 2015). In case surgery is performed, findings from pathology and direct culture of the removed suspected tissue or materials are considered the reference standard for the diagnosis. However, this is not always feasible and often the clinical diagnosis is settled by multidisciplinary consensus. This can be further affirmed by patient outcome during treatment and follow-up.

**Fig. 1:**
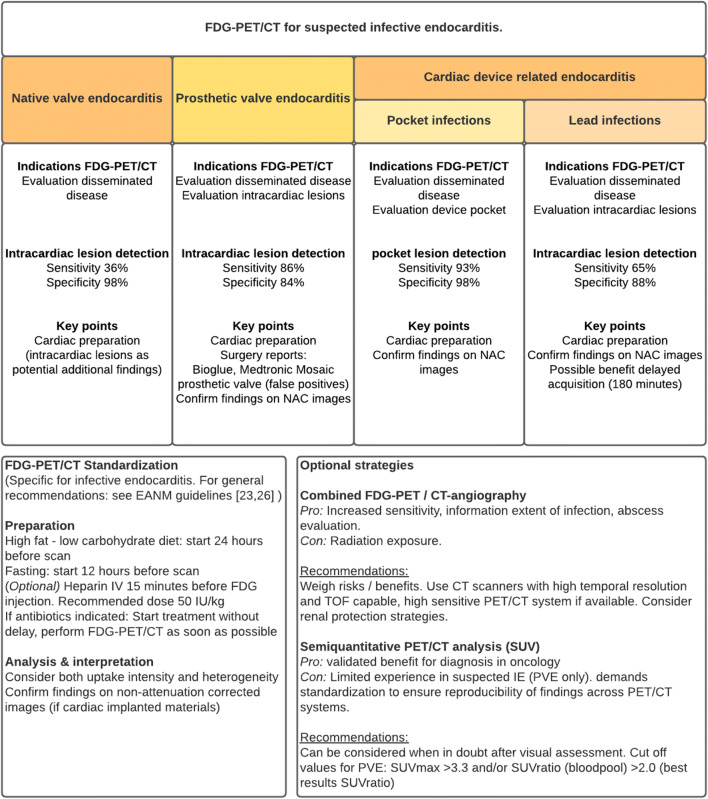
Overview of FDG-PET/CT indications for suspected IE and standardization strategies

Besides echocardiography, ^18^F-fluorodesoxyglucose with low-dose or contrast-enhanced computed tomography (FDG-PET/CT), cardiac CT, and leucocyte scintigraphy are the most frequently used imaging modalities for establishing IE [8]. Both most recent international guidelines (AHA/IDSA and ESC) leave many questions unanswered and a lot of room for interpretation. Ambiguity remains regarding the optimal use of these new imaging modalities: (i) Which imaging technique is recommended to be used first? (ii) For which patients are these techniques most suited? (iii) What is the optimal timing to apply imaging? and (iv) how can they best be performed and interpreted? This review focuses on FDG-PET/CT and gives an overview of indications for this technique in different patient groups, best practices concerning timing and approaches for standardization, to maximize its efficacy for clinical practice. A summary overview of the recommendations can be found in Fig. 1. These recommendations are based on available evidence and expert opinion. Recommendations based on specific guidelines are highlighted as such.

## Indications for FDG-PET/CT in Suspected IE

FDG-PET/CT can be used for two reasons when IE is suspected: it can either directly establish the presence of an infection in the endocardium or be used to find evidence for disseminated infection or portals of entry in IE disease. FDG-PET/CT is mainly applied when the diagnosis remains uncertain after other diagnostic tests are performed. Finding extracardiac foci of infection may help establish the diagnosis and can significantly impact treatment decisions [10]. When the main purpose of FDG-PET/CT is to evaluate the presence of IE in the endocardium, there are three main patient groups to differentiate between, those with (i) suspected native valve endocarditis (NVE), (ii) suspected prosthetic valve endocarditis (PVE), and (iii) cardiac device-related infective endocarditis (CDRIE). This distinction is important, since the presence of the different cardiac prosthetic materials affects ^18^F-FDG-PET/CT accuracy and its overall value for the diagnosis.

### Native Valve Endocarditis

The largest meta-analysis to date that focused on NVE specifically found that FDG-PET/CT showed a rather poor pooled sensitivity for the diagnosis, while pooled specificity was excellent: 36% and 99% respectively [11•, 12]. Because of its low sensitivity, negative intracardiac findings in FDG-PET/CT cannot be used to rule out the diagnosis in NVE. However, for most patients, there is an indication to perform FDG-PET/CT for detection of disseminated disease, as distant foci can help establishing the diagnosis. Additionally, if FDG PET/CT does show evidence for intracardiac infection in these patients, this is highly predictive for the diagnosis. This is especially relevant in patients in whom the diagnosis was not yet established with sufficient certainty by other investigations such as echocardiography and available clinical information. Therefore, our expert conclusion for clinical practice is that for suspected NVE, FDG-PET/CT can be performed to find evidence of disseminated disease, with appropriate patient preparation in order to maximize the chance of finding intracardiac infection as an additional finding (“by-catch”) with a very high specificity.

### Prosthetic Valve Endocarditis

For PVE, FDG-PET/CT has both high sensitivity and specificity for intracardiac infection. The most recent meta-analysis on this indication found a pooled sensitivity and specificity of 86% and 84%, respectively [12, 13•]. Furthermore, both sensitivity and specificity were higher in the more recent studies that were included in the meta-analysis, most likely as a result of improvements in patient preparation for and standardization of FDG-PET/CT and improvements in PET/CT camera systems. In conclusion, for this indication, the value of FDG is twofold, since it can provide both evidence of intracardiac infection and evidence of disseminated disease. This is especially important for this indication because echocardiography is often substantially hindered by prosthetic valve-related artefacts, leading to impairment of its diagnostic accuracy in this patient group [14].

Patients with combined prosthetic aortic valve implantation and ascending aorta replacement (so-called Bentall procedures) constitute a special group. Relatively little is known about the FDG-PET/CT findings for these indications. One study investigated the value for FDG-PET/CT for suspected Bentall infection in 39 patients [15]. The observed sensitivity and specificity were 86% and 80%, respectively, when only patients with focal FDG uptake or FDG uptake with soft tissue extension were considered positive for an infection. However, larger prospective studies are needed to validate these findings in this specific population. While the underlying aortic valve may be replaced by either a biological or mechanical valve, the ascending aorta is generally replaced by a synthetic material, usually Dacron®, which is associated with a risk of false-positive findings in similar large vessel vascular prostheses [16].

In both patient groups, the indication for a combination with ECG-gated CT angiography (CTA) should be considered with a rather low threshold, since abscess formation can be detected more reliably [12, 17], preferably in a “one-stop shopping” procedure (cf. section FDG PET/CT acquisition and reconstruction).

### Cardiac Device-Related Infective Endocarditis

For CDRIE, FDG-PET/CT has a high overall pooled sensitivity and specificity (87% and 94% respectively) according to the most recent meta-analysis on this indication [18]. However, CDRIE is a collective term that may apply both to pocket/generator infections and infections of the device leads. FDG-PET/CT performs markedly better for pocket infections than for lead infections: for pocket infections, pooled sensitivity and specificity were 93% and 98%, respectively, while for lead infections, sensitivity was poor (65%), although specificity was high (88%). Therefore, FDG-PET/CT can significantly attribute to the diagnosis of CDRIE-pocket infections. For CDRIE-lead infections however, it should be interpreted with caution and multidisciplinary consensus based on extensive clinical investigation is vital to correctly establish the diagnosis. There is some evidence that delayed image acquisition could increase FDG-PET/CT diagnostic accuracy in suspected CDRIE when intracardiac lead infection is suspected, though this study was small (n = 27) [19]. A recent meta-analysis in patients with suspected infection of a left ventricular assist device (LVAD) found a pooled sensitivity of 97% and pooled specificity of 99% in infections of the driveline, and a pooled sensitivity of 97% and pooled specificity of 93% for infection of the central device components [20]. For this indication, FDG-PET/CT has added value, although larger and prospective studies are necessary to provide more evidence.

## Timing of FDG-PET/CT

Literature about the optimal timing of FDG-PET/CT application is scarce. This applies both to the timing in the diagnostic workup in suspected IE and to the interval between cardiac surgery and FDG-PET/CT and suspected IE in the postoperative period. For the optimal timing in the diagnostic process, we recommend using transthoracic and transoesophageal echocardiography (TEE) first whenever feasible, since these are safe, fast, widely available, and cost-effective [12, 21]. TEE in particular yields a good diagnostic accuracy, which holds especially true when NVE is suspected [14, 21]. If FDG-PET/CT is indicated following echocardiography, it is recommended to perform it as soon as possible to allow for timely intervention when FDG-PET/CT confirms IE and to avoid false-negative findings as result of antibiotic treatment effect. Appropriate antibiotic treatment will over time lead to decreased inflammation, and C-reactive protein blood concentrations below 40 mg/L have been associated with false-negative FDG-PET/CT findings [22]. Since antibiotics are an integral part of IE treatment, the recommended course is to perform FDG-PET/CT before antibiotic treatment is initiated or as soon as possible promptly after, without delaying the start of antibiotic treatment.

Regarding cardiac surgery, the European Society of Cardiology guidelines recommend an empirical minimum interval of 3 months between the intervention and FDG-PET/CT before positive findings can be regarded as true positive [8]. This is a point of controversy, as EANM guidelines recommend a 1-month minimum interval [23]. There is also evidence that FDG-PET/CT is capable of showing true negative findings even within 1 month after implantation of prosthetic valves [22, 24, 25], while by contrast, false positives can still occur more than 3 months after, and possibly even up to 1 year after [22, 24]. False positives were strongly associated with use of a surgical adhesive: BioGlue (Cryolife Inc.) [22] and a specific bioprosthetic mitral valve model: the Medtronic Mosaic [23]. This valve model was associated with intense heterogeneous uptake 6 months after surgery that was characteristically absent 1 month after implantation. When the aforementioned confounders were not present in any of the included patients, only circular, homogenously increased FDG uptake was found in patients at 5, 12, and 52 weeks after PV implantation [25]. In conclusion, FDG-PET/CT results can be accurate as early as 1 month after heart valve replacement, but careful attention should be given to the surgical technique and materials that were used, up to and possibly beyond 1 year after surgery [22, 24, 25]. The same may apply to suspected CDRIE, but for this indication, data is lacking.

## Standardization

FDG-PET/CT standardization is important to ensure both repeatability of scan results and their reproducibility across different PET/CT systems, which is important to ensure maximum diagnostic accuracy and optimal (semi-) quantitative scan analysis. Standardization measures can be applied to patient preparation, scan acquisition, and scan analysis. Guidelines for standardization have been established by the European Association of Nuclear Medicine (EANM) and the European Association of Cardiovascular Imaging (EACVI) [26, 27••]. These general recommendations will be summarized in the following paragraphs, while the aforementioned guidelines can be consulted for an in-depth discussion.

### Patient Preparation

The main goal of patient preparation is to maximize tracer uptake in the target tissue against the background. Since FDG is a glucose analogue, it is taken up in non-affected tissue as well, dependent on their metabolic activity. This is especially the case in IE, where FDG is normally taken up in the myocardial cells. Patient preparation in IE is therefore aimed at limiting metabolic activity in the myocardium. The following measures are recommended before FDG-PET/CT in the setting of suspected IE, above the normal measures for patient preparation in FDG imaging [26]:

- Fasting and medication: Non-diabetic patients should not consume any foods or drinks besides water, preferably for at least 12 h prior to FDG-PET/CT. Medications can be taken as prescribed, with notable exception to corticosteroids, which should either be delayed until after FDG-PET/CT or be used at the lowest possible dose that is clinically feasible because of their interference with glucose metabolism [27••].
- High-fat, low-carbohydrate (HFLC) diet: the myocardium prefers free fatty acids over glucose for its metabolism. Therefore, using a high fatty acid and low-carbohydrate diet preceding FDG-PET/CT reduces cardiac glucose consumption. This will improve the target-to-background ratio resulting in optimal PET reading. Recommended is a 12-h HFLC diet, followed by the aforementioned 12-h fasting period [28–30].
- Heparin loading dose: Heparin causes free fatty acid release, which in turn decreases myocardial glucose uptake. There is some evidence that an intravenous heparin injection 15 min prior to FDG injection has an additive effect on physiological myocardial FDG uptake when used in conjunction with the HFLC diet [30], though other studies that evaluated heparin injection found variable results [31–33]. Because of the available, yet limited evidence for its additive value, when no contra-indications exist against the use of heparin, it may be considered as an adjunct to an HFLC diet. The recommended dose for heparin is 50 IU/kg for this indication [30].

### FDG PET/CT Acquisition and Reconstruction

The recommended administered activity for FDG-PET/CT is 2.5–5 MBq/kg according to EANM recommendations, which corresponds with 175 to 350 MBq for an adult weighing 70 kg [26]. The time interval between injection of FDG and start of the scan should be approximately 60 min. Documentation of the exact interval is necessary when semi-quantitative measurements, e.g., standardized uptake values (SUV), will be performed. The acceptable range for semi-quantitative analyses is 55–75 min [26]. For visual analysis, the exact interval between injection and FDG-PET/CT is of less importance and an interval of 60–90 min between injection and start of acquisition is acceptable [27••].

Because FDG remains trapped intracellularly for 3.5–4 h after injection, studies have been performed to evaluate whether delayed acquisition could increase FDG-PET/CT diagnostic accuracy. However, while there might be a potential benefit for CDRIE specifically [21], the increased risk of false positivity outweighs the benefit of a modest increase in sensitivity in PVE [34], and the value of delayed acquisition has not been evaluated in NVE.

**Fig. 2:**
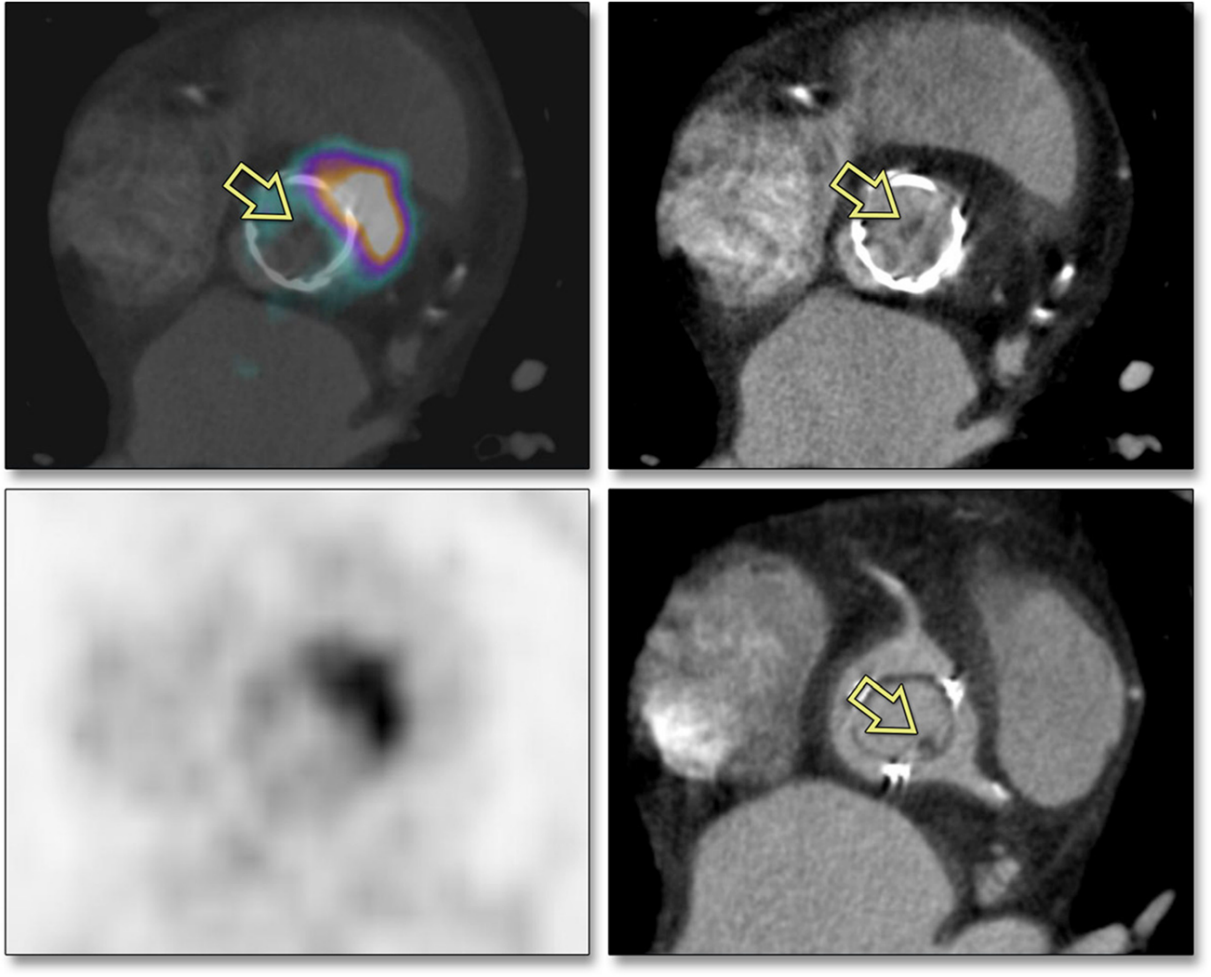
PET/CT angiography. Clinical case which demonstrates the additional value of combining FDG-PET and CTA. FDG-PET shows increased uptake at the edge of the prosthetic aortic valve, indicative of abscess formation (upper and lower left), but does not show the vegetation (*yellow arrows*) that is visible on CTA (diastolic phase: upper right; systolic phase: lower right). (This figure was published in the *Journal of the American College of Cardiology: Cardiovascular Imaging*, Vol 9, Scholtens AM, Swart LE, Verberne HJ, Tanis W, Lam MG, Budde RP, Confounders in FDG-PET/CT Imaging of Suspected Prosthetic Valve Endocarditis, Pages 1462-1465, Copyright Elsevier [2016]) [36]

For the diagnosis of IE, combining FDG-PET with CT angiography (CTA) leads to a combination of metabolic information of FDG-PET with a high anatomical reference, aiding in evaluation of solitary vegetations, soft tissue extension of infection, and/or potential abscesses. Studies using this combination are still scarce, but indicate towards benefit of the combination over single performed techniques [35]; see also Fig. 2 [36]. Care should be taken to minimize the risk of nephrotoxicity, as patients with IE are frequently at increased risk due to comorbidity and potentially nephrotoxic co-medication. Discontinuation of nephrotoxic co-medication, pre-hydration, and decreased contrast doses can minimize this risk. The radiation dose, which significantly increases when adding CTA to the procedure, is not a major issue anymore when using the newer PET and CT camera systems [27••, 37]. The increased sensitivity of FDG-PET/CTA should always be weighed against the associated risks for the patient and radiation reduction strategies should be employed whenever feasible.

### FDG PET/CT Analysis and Clinical Interpretation

There are both general and specific considerations for FDG-PET/CT assessment in the setting of suspected IE. As general considerations, verification of correct activity administration, image quality control, checks of blood glucose level, and PET/CT alignment are vital for a correct interpretation of FDG-PET/CT results. When increased myocardial physiological uptake is present, compliance to HFLC diet should be verified and reported upon. PET/CT images have to be evaluated in all 2D planes and in 3D maximum intensity projection (MIP) cine mode, taking into account both intensity of FDG uptake in the target lesion(s) and the pattern of FDG uptake. The uptake pattern of FDG is extremely important in defining whether there is IE. Homogeneous uptake mostly points to reactive inflammation and not infection. On the contrary, heterogeneous and/or (multi-) focal uptake points to an infection. Also, spread to surrounding soft tissue and/or metabolically active lymph nodes in the surrounding points to an infection [27••].

The imaging specialist should not only look to the attenuation-corrected images, but should also pay attention to the non-attenuated images, especially when a prosthetic valve or a cardiac device is present. Scatter or beam hardening artefacts caused by either prosthetic valves or cardiac-implanted devices can lead to false-positive findings. Increased FDG uptake should therefore always be confirmed on non-attenuation-corrected images to further confirm suspected prosthetic valve or device infection [27••].

As mentioned earlier, use of surgical adhesives (most specifically BioGlue) and one specific valve prosthesis model (Medtronic Mosaic) can lead to false-positive findings [22, 24]. Consequently, their presence should be taken into account during FDG-PET/CT evaluation. Therefore, the imaging specialist should be aware of the detailed reports of previous surgery. Scan results are ideally discussed within the multidisciplinary endocarditis team to assure findings are weighed appropriately relative to other clinical findings [27••].

Experience with semi-quantitative metrics using standardized uptake values (SUV) is extensive in oncology, but for IE, its use is less common. However, some promising results were found in a patient-control study when a SUVratio (defined as the SUVmax of the suspected lesion, divided by the SUVmean in the thoracic aorta) cutoff value >2.0 was used for diagnosing prosthetic valve endocarditis in EARL accredited, attenuation-corrected reconstructions. When patients with surgical adhesives and those with low inflammatory activity (CRP < 40 mg/L) were excluded, sensitivity and specificity were 100% and 91%, respectively, similar to visual analysis that showed a sensitivity and specificity of 91% and 95% respectively [22].

## Future Perspectives

New technical developments in PET/CT may lead to further improvement of FDG-PET/CT diagnostic accuracy and expansion of its clinical utility. In this section, new PET/CT acquisition protocols, hybrid imaging modalities, FDG-PET/CT treatment monitoring, and possibilities of artificial intelligence approaches are discussed. These are currently being evaluated and might emerge in clinical setting in the not too distant future.

### ECG Gating

One of the limitations of FDG-PET/CT is that it produces a static image. This potentially limits the interpretability of small intracardiac lesions because of cardiac motion artefacts and might be the explanation that isolated valve vegetations are associated with false-negative findings [22]. A potential solution to this could be the use of motion correction, e.g., through PET ECG gating. Elimination of motion artefacts could potentially increase PET/CT sensitivity, in particular for vegetations that are limited to valve leaflets or those attached to intracardiac leads, thereby increasing PET/CT sensitivity in NVE and CDRIE. ECG gating is possible in most current state-of-the-art PET/CT systems, and even options for dual gating, which include correction for respiration-related motion, are available. A remaining challenge is the trade-off between reduced motion artefacts and increased noise, resulting from gating-related loss of counts [38]. Data-driven approaches to minimize the loss of counts and the resulting image noise show promising results and may result in effective motion correction [38]. A proof of concept was shown for cardiac vitality PET [39]. Prospective studies are needed to evaluate whether these techniques have additive value over static PET/CT imaging in the setting of IE, since evidence for this indication, though promising, remains scarce [40].

### New Camera Systems

Current state-of-the-art digital PET/CT systems are capable of dealing with progressively lower photon counts, which has made dynamic PET/CT acquisition feasible. This combined with FDG uptake modelling allows for real-time assessment of FDG uptake rate in target tissues, potentially exposing differences in glucose metabolism impossible to detect on static PET/CT images. In oncology, dynamic FDG-PET/CT might be able to differentiate primary tumors from metastatic disease [41]. Potentially, the same could be applied to suspected IE for a better differentiation between infection and reactive or postsurgical inflammation.

Whole-body PET/CT systems incorporate multiple PET detector rings, which allows for a reduction of either scan time, radiation dose, or both, while giving a one-shot image of the whole body, potentially expanding the possibilities of the use of FDG-PET/CT to haemodynamically unstable patients, children, and pregnant women. It also allows further expansion of dynamic PET/CT abilities, leading to potentially significantly improved FDG-PET/CT applicability and sensitivity for a myriad of indications [42].

New PET/MRI systems are slowly finding more adoption around the world. This hybrid imaging modality allows for dynamic motion correction using MR data, excellent soft tissue evaluation, and advanced acquisitions to evaluate tissues for functional changes (e.g., late enhancement, diffusion-weighted imaging, and metabolic changes) [43]. This might be particularly useful for the evaluation of native valve endocarditis, for which PET/CT currently has limited sensitivity. For the PVE and CDRIE, a limitation of MRI is its susceptibility to artefacts caused by non-magnetic metals, which may significantly disrupt MRI-derived attenuation correction, while the risk of implanted device malfunction or lead overheating caused by radiofrequency interaction precludes some of these patients from undergoing MR examination completely.

### PET/CT-Guided Therapy

The information provided by FDG-PET/CT can not only be used for the diagnosis of IE, but potentially could also be used to monitor the effect of IE treatment, guiding therapeutic decision making, e.g., changing antibiotic dose, switching to a different therapeutic strategy, or deciding when treatment can safely be stopped. FDG-PET/CT is already adopted for this use in several oncological diseases, e.g., the treatment of multiple myeloma [44], and evidence is emerging that it could also be used for monitoring treatment of invasive fungal infections [45], tuberculosis [46], spondylodiscitis [47], and aortic graft infections [48]. For IE, to the best of our knowledge, no data currently exists. Considering the major challenges that remain for the treatment of IE, studies investigating the value of FDG-PET/CT for treatment monitoring in IE are urgently needed.

### Artificial Intelligence

Artificial intelligence and machine learning approaches are becoming increasingly incorporated in the field of nuclear medicine. The possibilities of artificial intelligence approaches range from data-driven noise reduction strategies [49], automated lesion delineation to advanced quantification possibilities. Currently, most progress has been described in oncology [50]. An exciting venue for future studies would be to evaluate whether artificial intelligence approaches can be used to distinguish between physiological uptake, reactive or postsurgical inflammation, and infection in suspected IE, which would dramatically increase the technique’s clinical utility.

### New Radiopharmaceuticals

Currently, FDG is the only PET radiotracer used in clinical practice for evaluation of IE. New radiotracers with bacteria-specific uptake are currently being evaluated, which could substantially improve PET/CT diagnostic accuracy. A systematic review by Auletta et al. evaluated some of the potential bacteria-specific candidates, e.g., ^18^F-Fluorosorbitol [51], ^18^F-Fluoromaltohexoase [52], and ^11^C-labeled para-aminobenzoic acid (PABA) [53], showed promising results for selectively binding specific bacteria, but currently, all these novel radiopharmaceuticals have only been validated in animal models [54]. Their clinical utility therefore still needs to be confirmed in human studies before they can be applied in clinical practice.

## Conclusion

FDG-PET/CT is a valuable tool for the evaluation of infective endocarditis. Its sensitivity is variable: excellent for the diagnosis of PVE and CDRIE-pocket infections, but poor for NVE and CDRIE-lead infections. The high specificity and ability to detect (clinically silent) foci of dissemination gives FDG-PET/CT a broad applicability and clinical utility for this challenging diagnosis. Standardization is of major importance for maximizing FDG-PET/CT diagnostic accuracy. It is recommended to perform the patient preparation and scan acquisition procedures in accordance with EANM guidelines. The clinical interpretation should be performed with attention to the clinical context, FDG-PET/CT image quality, recent cardiac surgery duration of antibiotic treatment prior to FDG-PET/CT, and confirmation of findings on NAC PET images. In the future, new developments in camera systems, developments in more specific tracers, and the use of artificial intelligence may substantially change the field of PET/CT imaging in patients with suspected IE.

## References

[1] Cahill TJ, Prendergast BD (2016). Infective endocarditis. Lancet..

[2] Jensen AD, Bundgaard H, Butt JH, Bruun NE, Voldstedlund M, Torp-Pedersen C, Gislason G, Iversen K, Chamat S, Dahl A, Køber L, Østergaard L, Fosbøl EL (2021). Temporal changes in the incidence of infective endocarditis in Denmark 1997-2017: a nationwide study. Int J Cardiol.

[3] Heredia-Rodríguez M, Hernández A, Bustamante-Munguira J, Álvarez FJ, Eiros JM, Castrodeza J, Tamayo E (2018). Evolution of the incidence, mortality, and cost of infective endocarditis in Spain between 1997 and 2014. J Gen Intern Med.

[4] van den Brink FS, Swaans MJ, Hoogendijk MG, Alipour A, Kelder JC, Jaarsma W, Eefting FD, Groenmeijer B, Kupper AJF, Ten Berg JM (2017). Increased incidence of infective endocarditis after the 2009 European Society of Cardiology guideline update: a nationwide study in the Netherlands. Eur Heart J Qual Care Clin Outcomes.

[5] Sunder S, Grammatico-Guillon L, Lemaignen A, Lacasse M, Gaborit C, Boutoille D, Tattevin P, Denes E, Guimard T, Dupont M, Fauchier L, Bernard L (2019). Incidence, characteristics, and mortality of infective endocarditis in France in 2011. PLoS One.

[6] Li JS, Sexton DJ, Mick N, Nettles R, Fowler VG, Ryan T, Bashore T, Corey GR (2000). Proposed modifications to the Duke criteria for the diagnosis of infective endocarditis. Clin Infect Dis.

[7] Prendergast BD (2004). Diagnostic criteria and problems in infective endocarditis. Heart..

[8] Habib G, Lancellotti P, Antunes MJ, Bongiorni MG, Casalta JP, Del Zotti F, Dulgheru R, El Khoury G, Erba PA, Iung B, Miro JM, Mulder BJ, Plonska-Gosciniak E, Price S, Roos-Hesselink J, Snygg-Martin U, Thuny F, Tornos Mas P, Vilacosta I (2015). Zamorano JL; ESC Scientific Document Group. 2015 ESC Guidelines for the management of infective endocarditis: The Task Force for the Management of Infective Endocarditis of the European Society of Cardiology (ESC). Eur Heart J.

[9] Baddour LM, Wilson WR, Bayer AS, Fowler VG, Tleyjeh IM, Rybak MJ, Barsic B, Lockhart PB, Gewitz MH, Levison ME, Bolger AF, Steckelberg JM, Baltimore RS, Fink AM, O’Gara P, Taubert KA (2015). American Heart Association Committee on Rheumatic Fever, Endocarditis, and Kawasaki Disease of the Council on Cardiovascular Disease in the Young, Council on Clinical Cardiology, Council on Cardiovascular Surgery and Anesthesia, and Stroke Council. Infective endocarditis in adults: diagnosis, antimicrobial therapy, and management of complications: a scientific statement for healthcare professionals from the American Heart Association. Circulation.

[10] Orvin K, Goldberg E, Bernstine H, Groshar D, Sagie A, Kornowski R, Bishara J (2015). The role of FDG-PET/CT imaging in early detection of extra-cardiac complications of infective endocarditis. Clin Microbiol Infect.

[11] Kamani CH, Allenbach G, Jreige M, Pavon AG, Meyer M, Testart N, Firsova M, Fernandes Vieira V, Boughdad S, Nicod Lalonde M, Schaefer N, Guery B, Monney P, Prior JO, Treglia G (2020). Diagnostic performance of 18F-FDG PET/CT in native valve endocarditis: systematic review and bivariate meta-analysis. Diagnostics (Basel).

[12] Gomes A, Glaudemans AWJM, Touw DJ, van Melle JP, Willems TP, Maass AH, Natour E, Prakken NHJ, Borra RJH, van Geel PP, Slart RHJA, van Assen S, Sinha B (2017). Diagnostic value of imaging in infective endocarditis: a systematic review. Lancet Infect Dis.

[13] TKM W, Sánchez-Nadales A, Igbinomwanhia E, Cremer P, Griffin B, Xu B (2020). Diagnosis of infective endocarditis by subtype using 18F-fluorodeoxyglucose positron emission tomography/computed tomography: a contemporary meta-analysis. Circ Cardiovasc Imaging.

[14] Zoghbi WA, Chambers JB, Dumesnil JG, Foster E, Gottdiener JS, Grayburn PA, Khandheria BK, Levine RA, Marx GR, Miller FA, Nakatani S, Quiñones MA, Rakowski H, Rodriguez LL, Swaminathan M, Waggoner AD, Weissman NJ (2009). Zabalgoitia M; Recommendations for evaluation of prosthetic valves with echocardiography and Doppler ultrasound. J Am Soc Echocardiogr.

[15] Lucinian YA, Lamarche Y, Demers P, Martineau P, Harel F, Pelletier-Galarneau M (2020). FDG-PET/CT for the detection of infection following aortic root replacement surgery. JACC Cardiovasc Imaging.

[16] Keidar Z, Pirmisashvili N, Leiderman M, Nitecki S, Israel O (2014). 18F-FDG uptake in noninfected prosthetic vascular grafts: incidence, patterns, and changes over time. J Nucl Med.

[17] Gomes A, van Geel PP, Santing M, Prakken NHJ, Ruis ML, van Assen S (2018). Imaging infective endocarditis: adherence to a diagnostic flowchart and direct comparison of imaging techniques. J Nucl Cardiol.

[18] Liao L, Kong DF, Samad Z, Pappas PA, Jollis JG, Lin SS, Wang A, Fowler VG, Chu VH, Sexton DJ, Corey GR, Cabell CH (2008). Echocardiographic risk stratification for early surgery with endocarditis: a cost-effectiveness analysis. Heart..

[19] Juneau D, Golfam M, Hazra S, Zuckier LS, Garas S, Redpath C, Bernick J, Leung E, Chih S, Wells G (2017). Positron emission tomography and single-photon emission computed tomography imaging in the diagnosis of cardiac implantable electronic device infection: a systematic review and meta-analysis. Circ Cardiovasc Imaging.

[20] Ten Hove D, Treglia G, Slart RHJA, Damman K, Wouthuyzen-Bakker M, Postma DF, et al. The value of 18F-FDG PET/CT for the diagnosis of device-related infections in patients with a left ventricular assist device: a systematic review and meta-analysis. Eur J Nucl Med Mol Imaging. 2020. 10.1007/s00259-020-04930-8 PMC7835315 32594196

[21] Leccisotti L, Perna F, Lago M, Leo M, Stefanelli A, Calcagni ML, Pelargonio G, Narducci ML, Bencardino G, Bellocci F, Giordano A (2014). Cardiovascular implantable electronic device infection: delayed vs standard FDG PET-CT imaging. J Nucl Cardiol.

[22] Swart LE, Gomes A, Scholtens AM, Sinha B, Tanis W, Lam MGEH, van der Vlugt MJ, Streukens SAF, Aarntzen EHJG, Bucerius J, van Assen S, Bleeker-Rovers CP, van Geel PP, Krestin GP, van Melle JP, Roos-Hesselink JW, Slart RHJA, Glaudemans AWJM, Budde RPJ (2018). Improving the diagnostic performance of 18F-fluorodeoxyglucose positron-emission tomography/computed tomography in prosthetic heart valve endocarditis. Circulation..

[23] Jamar F, Buscombe J, Chiti A, Christian PE, Delbeke D, Donohoe KJ, Israel O, Martin-Comin J, Signore A (2013). EANM/SNMMI guideline for 18F-FDG use in inflammation and infection. J Nucl Med.

[24] Roque A, Pizzi MN, Fernández-Hidalgo N, Permanyer E, Cuellar-Calabria H, Romero-Farina G, Ríos R, Almirante B, Castell-Conesa J, Escobar M, Ferreira-González I, Tornos P, Aguadé-Bruix S (2020). Morpho-metabolic post-surgical patterns of non-infected prosthetic heart valves by [18F]FDG PET/CTA: “normality” is a possible diagnosis. Eur Heart J Cardiovasc Imaging.

[25] Wahadat AR, Tanis W, Scholtens AM, Bekker M, Graven LH, Swart LE, et al. Normal imaging findings after aortic valve implantation on 18F-Fluorodeoxyglucose positron emission tomography with computed tomography. J Nucl Cardiol. 2020. 10.1007/s12350-019-02025-y PMC8648629 31975327

[26] Boellaard R, Delgado-Bolton R, Oyen WJ, Giammarile F, Tatsch K, Eschner W, Verzijlbergen FJ, Barrington SF, Pike LC, Weber WA, Stroobants S, Delbeke D, Donohoe KJ, Holbrook S, Graham MM, Testanera G, Hoekstra OS, Zijlstra J, Visser E, Hoekstra CJ, Pruim J, Willemsen A, Arends B, Kotzerke J, Bockisch A, Beyer T, Chiti A, Krause BJ (2015). European Association of Nuclear Medicine (EANM). FDG PET/CT: EANM procedure guidelines for tumour imaging: version 2.0. Eur J Nucl Med Mol Imaging.

[27] RHJA S, AWJM G, Gheysens O, Lubberink M, Kero T, Dweck MR, Habib G, Gaemperli O, Saraste A, Gimelli A, Georgoulias P, Verberne HJ, Bucerius J, Rischpler C, Hyafil F, Erba PA (2020). 4Is cardiovascular imaging: a joint initiative of the European Association of Cardiovascular Imaging (EACVI); European Association of Nuclear Medicine (EANM). Procedural recommendations of cardiac PET/CT imaging: standardization in inflammatory-, infective-, infiltrative-, and innervation (4Is)-related cardiovascular diseases: a joint collaboration of the EACVI and the EANM. Eur J Nucl Med Mol Imaging.

[28] Williams G, Kolodny GM. Suppression of myocardial 18F-FDG uptake by preparing patients with a high-fat, low-carbohydrate diet. AJR Am J Roentgenol. 2008;190(2):W151–6. 10.2214/AJR.07.2409 18212199

[29] Balink H, Hut E, Pol T, Flokstra FJ, Roef M (2011). Suppression of 18F-FDG myocardial uptake using a fat-allowed, carbohydrate-restricted diet. J Nucl Med Technol.

[30] Scholtens AM, van den Berk AM, van der Sluis NL, Esser JP, Lammers GK, de Klerk JMH, Lam MGEH, Verberne HJ (2020). Suppression of myocardial glucose metabolism in FDG PET/CT: impact of dose variation in heparin bolus pre-administration. Eur J Nucl Med Mol Imaging.

[31] Larson SR, Pieper JA, Hulten EA, Ficaro EP, Corbett JR, Murthy VL, Weinberg RL (2020). Characterization of a highly effective preparation for suppression of myocardial glucose utilization. J Nucl Cardiol.

[32] Manabe O, Yoshinaga K, Ohira H, Masuda A, Sato T, Tsujino I, Yamada A, Oyama-Manabe N, Hirata K, Nishimura M, Tamaki N (2016). The effects of 18-h fasting with low-carbohydrate diet preparation on suppressed physiological myocardial (18)F-fluorodeoxyglucose (FDG) uptake and possible minimal effects of unfractionated heparin use in patients with suspected cardiac involvement sarcoidosis. J Nucl Cardiol.

[33] Morooka M, Moroi M, Uno K, Ito K, Wu J, Nakagawa T, Kubota K, Minamimoto R, Miyata Y, Okasaki M, Okazaki O, Yamada Y, Yamaguchi T, Hiroe M (2014). Long fasting is effective in inhibiting physiological myocardial 18F-FDG uptake and for evaluating active lesions of cardiac sarcoidosis. EJNMMI Res.

[34] Scholtens AM, Swart LE, Verberne HJ, Budde RPJ, Lam MGEH (2018). Dual-time-point FDG PET/CT imaging in prosthetic heart valve endocarditis. J Nucl Cardiol.

[35] Wahadat AR, Tanis W, Swart LE, Scholtens A, Krestin GP, van Mieghem NMDA, et al. Added value of ^18^F-FDG-PET/CT and cardiac CTA in suspected transcatheter aortic valve endocarditis. J Nucl Cardiol. 2019. 10.1007/s12350-019-01963-x PMC8648682 31792918

[36] Scholtens AM, Swart LE, Verberne HJ, Tanis W, Lam MG, Budde RP (2016). Confounders in FDG-PET/CT imaging of suspected prosthetic valve endocarditis. JACC Cardiovasc Imaging.

[37] Faure ME, Swart LE, Dijkshoorn ML, Bekkers JA, van Straten M, Nieman K, Parizel PM, Krestin GP, Budde RPJ (2018). Advanced CT acquisition protocol with a third-generation dual-source CT scanner and iterative reconstruction technique for comprehensive prosthetic heart valve assessment. Eur Radiol.

[38] Rubeaux M, Doris MK, Alessio A, Slomka PJ (2017). Enhancing cardiac PET by motion correction techniques. Curr Cardiol Rep.

[39] Slomka PJ, Rubeaux M, Le Meunier L, Dey D, Lazewatsky JL, Pan T, Dweck MR, Newby DE, Germano G, Berman DS (2015). Dual-gated motion-frozen cardiac PET with flurpiridaz F 18. J Nucl Med.

[40] Boursier C, Duval X, Bourdon A, Imbert L, Mahida B, Chevalier E, Claudin M, Hoen B, Goehringer F, Selton-Suty C, Roch V, Lamiral Z, Humbert O, Rouzet F, Marie PY (2020). AEPEI-TEPvENDO study group. ECG-gated cardiac FDG PET acquisitions significantly improve detectability of infective endocarditis. JACC Cardiovasc Imaging.

[41] Yamanaka M, Shinya T, Otomi Y, Kubo M, Arai Y, Toba H, Bando Y, Otsuka H, Harada M (2020). Semiquantitative assessment of fluorodeoxyglucose uptake in primary tumours on dynamic PET/computed tomography for lymph node metastasis evaluation in patients with lung cancer: a prospective study. Nucl Med Commun.

[42] Fu F, Li X, Wu Y, Xu J, Bai Y, Gao Y, Wang Z, Zhang W, Wei W, El Fakhri G, Shao F, Wang M (2021). Total-body dynamic PET/CT of micro-metastatic lymph node in a patient with lung cancer. Eur J Nucl Med Mol Imaging.

[43] Sollini M, Berchiolli R, Kirienko M, Rossi A, Glaudemans AWJM, Slart R, Erba PA (2018). PET/MRI in infection and inflammation. Semin Nucl Med.

[44] Cavo M, Terpos E, Nanni C, Moreau P, Lentzsch S, Zweegman S, Hillengass J, Engelhardt M, Usmani SZ, Vesole DH, San-Miguel J, Kumar SK, Richardson PG, Mikhael JR, da Costa FL, Dimopoulos MA, Zingaretti C, Abildgaard N, Goldschmidt H, Orlowski RZ, Chng WJ, Einsele H, Lonial S, Barlogie B, Anderson KC, Rajkumar SV, BGM D, Zamagni E (2017). Role of 18F-FDG PET/CT in the diagnosis and management of multiple myeloma and other plasma cell disorders: a consensus statement by the International Myeloma Working Group. Lancet Oncol.

[45] Ankrah AO, Span LFR, Klein HC, de Jong PA, Dierckx RAJO, Kwee TC, Sathekge MM, Glaudemans AWJM (2019). Role of FDG PET/CT in monitoring treatment response in patients with invasive fungal infections. Eur J Nucl Med Mol Imaging.

[46] Malherbe ST, Chen RY, Dupont P, Kant I, Kriel M, Loxton AG, Smith B, Beltran CGG, van Zyl S, McAnda S, Abrahams C, Maasdorp E, Doruyter A, Via LE, Barry CE, Alland D, Richards SG, Ellman A, Peppard T, Belisle J, Tromp G, Ronacher K, Warwick JM, Winter J, Walzl G (2020). Quantitative 18F-FDG PET-CT scan characteristics correlate with tuberculosis treatment response. EJNMMI Res.

[47] Righi E, Carnelutti A, Muser D, Di Gregorio F, Cadeo B, Melchioretto G, Merelli M, Alavi A, Bassetti M (2020). Incremental value of FDG-PET/CT to monitor treatment response in infectious spondylodiscitis. Skelet Radiol.

[48] Husmann L, Ledergerber B, Anagnostopoulos A, Stolzmann P, Sah BR, Burger IA, Pop R, Weber A, Mayer D, Rancic Z (2018). Hasse B; VASGRA Cohort Study. The role of FDG PET/CT in therapy control of aortic graft infection. Eur J Nucl Med Mol Imaging.

[49] Cui J, Gong K, Guo N, Wu C, Meng X, Kim K, Zheng K, Wu Z, Fu L, Xu B, Zhu Z, Tian J, Liu H, Li Q (2019). PET image denoising using unsupervised deep learning. Eur J Nucl Med Mol Imaging.

[50] Seifert R, Weber M, Kocakavuk E, Rischpler C, Kersting D (2021). Artificial intelligence and machine learning in nuclear medicine: future perspectives. Semin Nucl Med.

[51] Li J, Zheng H, Fodah R, Warawa JM, Ng CK (2018). Validation of 2-18F-fluorodeoxysorbitol as a potential radiopharmaceutical for imaging bacterial infection in the lung. J Nucl Med.

[52] Takemiya K, Ning X, Seo W, Wang X, Mohammad R, Joseph G, Titterington JS, Kraft CS, Nye JA, Murthy N, Goodman MM, Taylor WR (2019). Novel PET and near infrared imaging probes for the specific detection of bacterial infections associated with cardiac devices. JACC Cardiovasc Imaging.

[53] Mutch CA, Ordonez AA, Qui H, Parker M, Bambarger LE, Villanueva-Meyer JE, Blecha J, Carroll V, Taglang C, Flavell R (2018). [11C]Para-aminobenzoic acid: a positron emission tomography tracer targeting bacteria-specific metabolism. ACS Infect Dis.

[54] Auletta S, Varani M, Horvat R, Galli F, Signore A, Hess S (2019). PET radiopharmaceuticals for specific bacteria imaging: a systematic review. J Clin Med.

